# Do you truly feel pleasant? The effect of question formulations on affective responses to acute exercise

**DOI:** 10.3389/fpsyg.2025.1663494

**Published:** 2025-09-08

**Authors:** Qi Zhang

**Affiliations:** School of Psychology, Beijing Sport University, Beijing, China

**Keywords:** acute exercise, affective responses, question formulations, Feeling Scale, heart rate variability

## Abstract

The current study used the Feeling Scale, developed by Hardy and Rejeski, to examine how question formulations affect affective responses during acute exercise. A within-group experimental design of 3 (question formulations) × 6 (measurement time) was used. The three types of question formulations were positive, negative, and neutral. The measurement time was divided into three categories: prior to the beginning of the exercise, at 5-min intervals during the exercise, and immediately following the cool-down, resulting in a total of 6 measures. Participants’ heart rate was tracked throughout, and affective responses were evaluated using one question formulation per exercise, in a random order. We recruited 21 college students to complete three 30-min moderate-intensity running sessions (2 days apart). A repeated-measures ANOVA treated question formulations and measurement time as independent variables. Results showed that: (1) When ratings of affective responses were the dependent variable, the main effect of measurement time (*F*_(1.726, 34.522)_ = 8.043, η*_p_*^2^ = 0.287, *p* < 0.01) and question formulation (*F*_(1.928, 38.567)_ = 6.382, η*_p_*^2^ = 0.242, *p* < 0.01) were significant, and the interaction between the two was significant (*F*_(3.873, 77.461)_ = 3.552, η*_p_*^2^ = 0.151, *p* < 0.05). Simple effects analyses showed that affective responses in positive question formulations were superior to those in negative, but not significantly distinct from those in neutral. Neutral question formulations got better affective responses than negative question formulations. (2) For SDNN and RMSSD, the main effect of measurement time was significant (*F*_(2.514, 50.270)_ = 30.739, η*_p_*^2^ = 0.606, *p* < 0.001; *F*_(2.115, 42.307)_ = 44.041, η*_p_*^2^ = 0.688, *p* < 0.001). Acute exercise led to notable drops in SDNN and RMSSD, indicating acute exercise significantly reduced both parameters. Neither the main effect of question formulation nor the interaction was significant. Question formulations influenced affective responses: positive and neutral formulations elicited better affective responses than negative formulations.

## Introduction

Despite an extensive amount of research demonstrating the positive impact of regular physical activity on lowering the risk of cardiovascular diseases, diabetes, cancer, and stroke (Lee et al., 2003; Warburton, 2006), a significant portion of the population remains insufficiently engaged in physical activity, failing to meet the World Health Organization’s (WHO) recommended guidelines of 150–300 min per week of moderate-intensity or 75–150 min per week of high-intensity physical activity (World Health Organization, 2020). Hence, it’s crucial to figure out how to encourage people to engage in and adhere with physical activity. The theory of hedonic motivation suggests that the affective experience during physical activity will influence future physical activity participation (Williams, 2018). It states that pleasure or displeasure experienced during a behavior is associated with future hedonic motivation for that behavior (Williams, 2018). It is apparent that enhancing affective responses to exercise constitutes a viable strategy for promoting engagement in physical activity or exercise.

Researchers have conducted numerous studies to enhance affective responses to exercise. More positive and less negative affect experienced during physical activity was associated with more participation in the future (Liao et al., 2017). In the study conducted by Kwan et al. (2017), participants were instructed to read exercise descriptions that were either positive or negative before engaging in the exercise. The study revealed that the use of positive induction elicited more favorable affective responses in comparison to negative induction. The same outcomes were discovered in the Wang et al.’s (2022) study. Compared to negative induction, positive pre-exercise induction improved participants’ affective responses during and after exercise. It is clear that information, whether positive or negative, could impact an individual’s affective responses during or after exercise.

Researchers frequently use the single-item Feeling Scale (FS) to assess affective responses (Decker and Ekkekakis, 2017; Oliveira et al., 2023; Zenko et al., 2020). “How do you feel right now?” is the item. There are no positive or negative inducements in the question formulation. Researchers often take measurements every 5 min during exercise to obtain affective responses. As previously discussed (Kwan et al., 2017; Wang et al., 2022), affective responses can be influenced by positive or negative information given to individuals before or during exercise. Giving individuals positive or negative instructions before or during exercise can have an impact on their affective responses. But no researcher has examined whether people differ in how they react affectively when questions that simply measure affective response items are framed positively or negatively, and whether people respond differently when they are not elicited by other manipulations. Hence, the current study developed positive and negative question formulations derived from the FS of the initial neutral question formulation. The objective of this study is to examine possible differences in affective responses as measured by different question formulations during acute exercise.

Heart rate variability (HRV) is defined as the variation in time intervals between successive heartbeats (Lin et al., 2016). A lower HRV indicates reduced parasympathetic control of the heart, suggesting that individuals have difficulty managing emotions and coping with stress. Less physically active individuals reported reduced affective valence and arousal when viewing photos connected to exercise (Schinkoeth et al., 2019). Also, HRV has dropped. It could be of particular interest for research on affect (Laborde et al., 2017). HRV can be a valid physiological indicator of an individual’s affective state during and after exercise (Li et al., 2021). It can be used as an index for the regulation of affect via prefrontal-subcortical structures (Thayer et al., 2012). As a result, we will track HRV during exercise in an attempt to offer a psychophysiological explanation for affective responses.

In summary, the effects of various question formulations on affective responses during acute exercise were the main focus of this study. The study did not include any further interventions or procedures to induce or initiate affective responses in participants. The only variation was in the wording of the measurement item’s question. Participants’ HRV during exercise was also continuously recorded. The current study put forth the hypothesis that, in contrast to neutral or negative question formulations, participants would report greater positive affective responses both during and after exercise when positive question formulations were used.

## Methods

### Participants

The sample size was determined through power calculations conducted with G*Power v.3.1 (Faul et al., 2009). To achieve an effect size (*f*) of 0.25 with a power (1-β) of 0.80 at the significance level (α) of 0.05 in a repeated measures ANOVA considering the within factors (number of measurements = 18, number of groups = 1), a sample size of 10 was sufficient. To ensure the robustness of the results, this study recruited 26 college students. Two of the participants decided not to continue with the experiment. In the middle of the experiment, three participants withdrew. Hence, the total number of valid participants was 21 (3 males, 18 females; *M*_age_ = 19.619 ± 1.857, *M*_BMI_ = 21.785 ± 2.658 kg/m^2^). Prior to the formal experiment, every participant signed informed consent. At any point, participants were free to leave the experiment. The Ethics Committee of the School of Psychology at Beijing Sport University approved the research protocol.

### Design

A 3 (question formulations) × 6 (measurement time) within-group experimental design was employed. The dependent variable was affective response ratings. Three types of question formulations were identified: neutral, negative, and positive. Measurement times were taken before the start of exercise (T0), at minutes 5 during exercise (T1), at minutes 10 during exercise (T2), at minutes 15 during exercise (T3), at minutes 20 during exercise (T4), and a cool-down period (5 min after exercise, T5).

### Materials

#### Feeling scale

The affective responses of individuals to acute exercise were measured with FS (Hardy and Rejeski, 1989). The question was formulated neutrally and asked, “How do you feel right now?.” FS is an 11-point single-item Likert scale, ranging from −5 (extremely bad) to 5 (extremely good), with 0 representing neutral.

#### Revised Feeling Scale

On the basis of FS (Hardy and Rejeski, 1989), we developed formulations for both positive and negative questions. The positive question formulation was “How pleasant do you feel right now?,” ranging from −5 (extremely unpleasant) to 5 (extremely pleasant), with 0 being neither good nor bad. The negative question formulation was “How bad do you feel right now?,” ranging from −5 (extremely bad) to 5 (extremely not bad), with 0 representing neutral.

#### The rating of perceived exertion scale

Rating of perceived exertion (RPE) was used to assess perceived physical exertion during the running session (Borg, 1982). RPE is measured on a 15-point scale ranging from 6 (no exertion) to 20 (maximum exertion), with a score of 12 ~ 14 indicating the moderate intensity. Measurement times included 5 min during exercise (T1), 10 min during exercise (T2), 15 min during exercise (T3), and 20 min during exercise (T4).

#### Heart rate variability

We used Polar watch (Grit X) and a heart rate belt to track HRV. Two time-domain indicators were extracted from HRV signals: the standard deviation of normal-to-normal intervals (SDNN) and the root mean square of successive differences (RMSSD). SDNN reflects overall HRV magnitude and indicates sympathetic and parasympathetic nervous system excitability. RMSSD is an indicator of parasympathetic activity and is suitable as a measure of short-term HRV status. Kubios HRV software was used to analyze the HRV data (Tarvainen et al., 2014). Artifact correction was performed through an initial exclusion of RR intervals exhibiting greater than 25% deviation from both adjacent intervals. These aberrant intervals were subsequently replaced utilizing a traditional spline interpolation method, consistent with established methodologies, in conjunction with the application of the medium-grade filter available in the Kubios HRV Standard software (Shaffer and Ginsberg, 2017). Subsequently, a smoothness prior approach, employing a lambda value of 500, was implemented to remove spurious low-frequency baseline trend elements (Tarvainen et al., 2014). While the low-frequency/high-frequency (LF/HF) ratio theoretically reflects sympathovagal balance, rapid breathing during exercise may cause an upward shift in the HF band, overlapping with the LF band and potentially distorting calculations. Therefore, the LF/HF ratio was not selected for analysis in this study.

### Procedure

**Figure 1:**
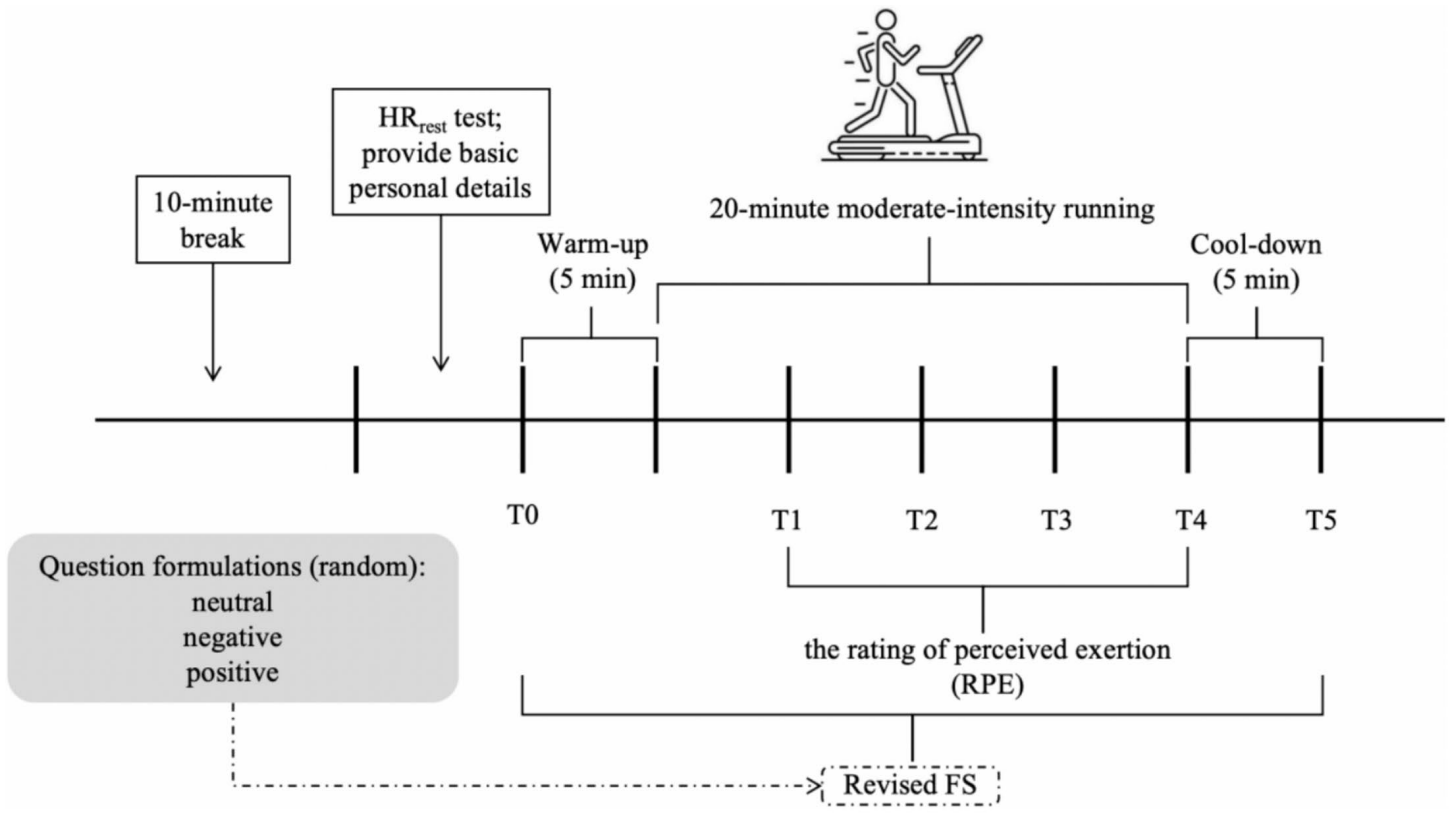
Overview of study protocol.

In order to track their HRV throughout the entire session, participants wore the heart rate belt tight to their chest and linked it with a Polar watch after a 10-min break when they arrived at the lab. Participants observed HR_rest_, which computes HR intervals at moderate exercise intensity, while they sat motionless in front of a viewpoint-only computer screen for 5 min. Following the HR_rest_ test, participants were asked to provide some basic personal details, such as their height, weight, and birth date. The participants were then prepared to run on the treadmill, and affective responses were recorded before the exercise began. After the running session began, measures of affective responses and perceived exertion were recorded every 5-min. Following a 5-min cool-down period, which followed the run, assessments of the participants’ immediate post-exercise affective responses were made (see Figure 1).

Participants were required to come to the lab a total of three times. Complete 30-min moderate-intensity running sessions at each session (2 days apart) while wearing a heart rate belt and Polar watch. During each running session, participants were asked to evaluate their perception of exertion, and measures of affective responses to different question formulations (positive, negative, or neutral) were obtained. Affective response measures comprise a randomized sequence of question formulations. Following the completion of the third run, participants received deserved rewards.

### Data analysis

Statistical analyses included descriptive statistics (means and standard deviations) for all variables. We did a 3 (question formulations: positive, negative, neutral) × 6 (measurement time: T0, T1, T2, T3, T4, T5) repeated-measures ANOVA with the ratings of affective responses, SDNN, and RMSSD as the dependent variables, respectively, to see how different question formulations affected affective responses and HRV during acute exercise. The Bartlett’s test of sphericity showed a significant result (*p* < 0.05), indicating that the assumption of sphericity was violated. As a result, the Greenhouse–Geisser correction was applied to fix this violation. Significant level was set at 0.05. SPSS 26.0 was used to analyze all the data.

## Results

### RPE

Table 1 showed the descriptive statistics for the affective response ratings and RPE of the participants. Results showed that the main effect of question formulation was not significant (*F*_(2, 19)_ = 0.073, η*_p_*^2^ = 0.008, *p* > 0.05), participants’ RPE remained consistent with moderate intensity across conditions.

**Table 1:** Descriptive statistics of affective response ratings and RPE (*M* ± *SD*).

Ratings	T0	T1	T2	T3	T4	T5
FS_pos_	1.667 ± 2.331	1.428 ± 2.378	1.238 ± 2.256	1.524 ± 2.379	1.714 ± 2.411	2.809 + 1.692
FS_neg_	2.524 ± 2.657	0.905 ± 2.700	0.476 ± 2.695	0.286 ± 2.217	0.286 ± 2.533	1.952 ± 2.291
FS_neu_	2.571 ± 1.568	2.286 ± 1.419	2.000 ± 1.612	1.667 ± 1.983	1.667 ± 2.033	2.905 ± 1.411
RPE_pos_	–	11.190 ± 1.365	12.190 ± 1.965	12.762 ± 1.921	12.857 ± 1.195	–
RPE_neg_	–	11.190 ± 1.940	12.143 ± 1.834	12.524 ± 1.632	12.762 ± 1.895	–
RPE_neu_	–	11.286 ± 1.765	12.143 ± 1.621	12.524 ± 1.537	12.905 ± 1.091	–

FS, feeling scale; RPE, ratings of perceived exertion. FS_pos_, FS_neg_, and FS_neu_ indicate affective responses measured when the question formulations were positive, negative, and neutral, respectively. RPE_pos_, RPE_neg_, and RPE_neu_ indicate perceived exertion ratings measured when the question formulations were positive, negative, and neutral, respectively.

### FS

Results showed that the main effect of measurement time (*F*_(1.726, 34.522)_ = 8.043, η*_p_*^2^ = 0.287, *p* < 0.01) and question formulation (*F*_(1.928, 38.567)_ = 6.382, η*_p_*^2^ = 0.242, *p* < 0.01) were both significant, as was the interaction between the two (*F*_(3.873, 77.461)_ = 3.552, η*_p_*^2^ = 0.151, *p* < 0.05). According to simple effects analysis, affective responses were found to be better in positive question formulations compared to negative question formulations, but they did not differ substantially from neutral question formulations. Neutral question formulations elicited more positive affective responses than negative ones.

**Figure 2:**
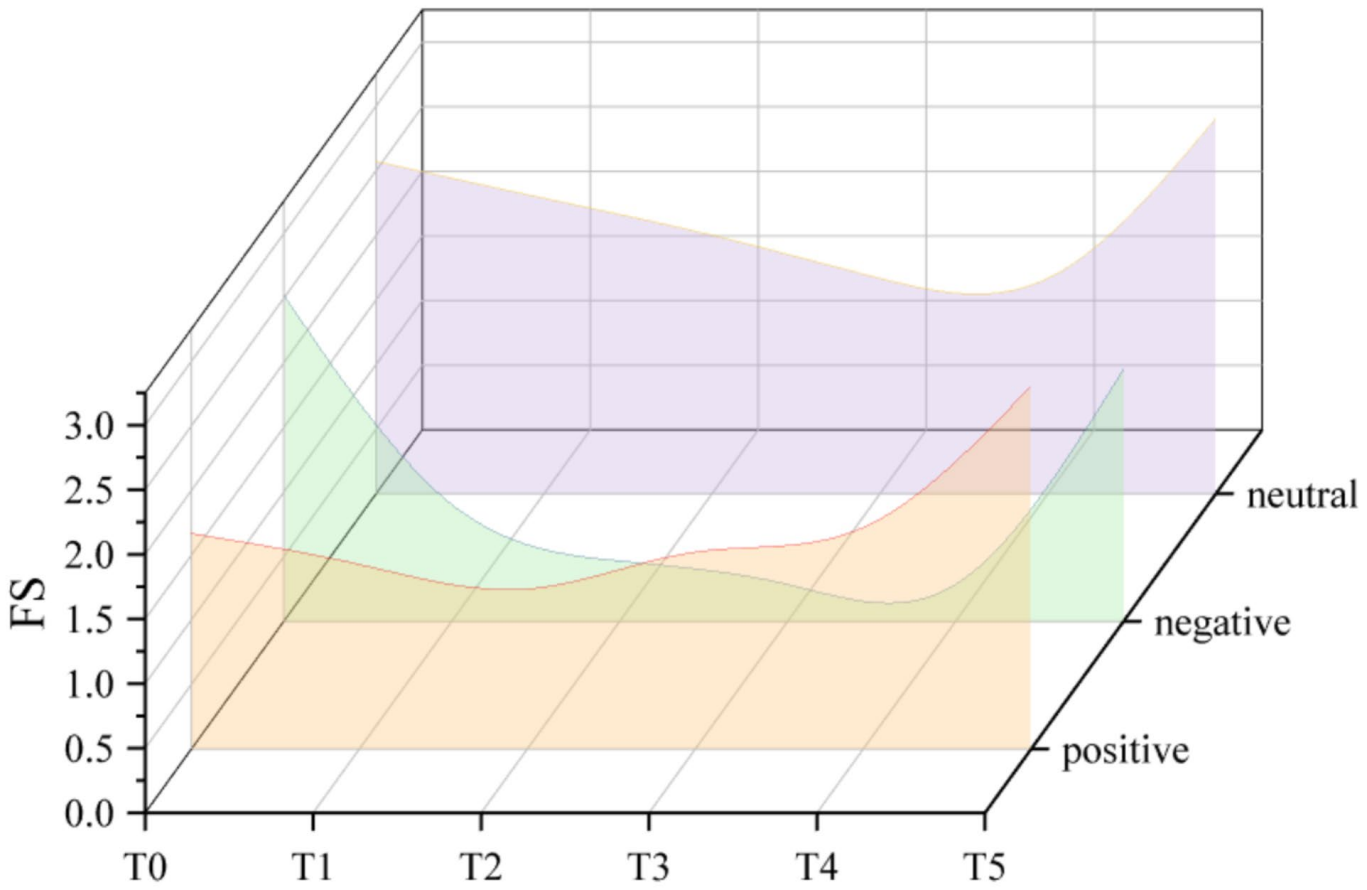
Trend of changes in affective responses under different question formulations.

Participants’ affective response ratings differed between measurement times when the questions were formulated differently, as Table 2 and Figure 1 illustrate. Affective rebound was observed during the last assessment of affective responses (immediately after the end of cooling). When the question formulation was positive, affective responses from participants were considerably higher at T5 than at T1 through T4. When the question formulation was negative, affective responses from participants were considerably higher at T5 than at T3 through T4. When the question formulation was neutral, affective responses from participants were considerably higher at T5 than at T2 through T4. While there was no statistically significant difference in affective response ratings during the final measurement, Figure 2 illustrates that the mean affective response ratings was lower than the pre-exercise baseline only when the question formulation was negative.

**Table 2:** Comparison of affective response ratings at different measurement times.

Measurement time	Question formulation		Standard deviation	*p*	95% CI
T0	Positive	Negative	0.416	0.149	[−1.94, 0.23]
	Positive	Neutral	0.337	**0.042**	**[−1.78, −0.03]**
	Negative	Neutral	0.480	1.000	[−1.30, 1.20]
T1	Positive	Negative	0.450	0.593	[−0.65, 1.70]
	Positive	Neutral	0.449	0.197	[−2.21, 0.31]
	Negative	Neutral	0.514	**0.042**	**[−2.72, −0.04]**
T2	Positive	Negative	0.447	0.280	[−0.40, 1.95]
	Positive	Neutral	0.344	0.112	[−1.66, 0.14]
	Negative	Neutral	0.515	**0.023**	**[−2.86, −0.18]**
T3	Positive	Negative	0.483	0.054	[−0.02, 2.50]
	Positive	Neutral	0.311	0.957	[−0.95, 0.67]
	Negative	Neutral	0.450	**0.018**	**[−2.55, −0.21]**
T4	Positive	Negative	0.505	**0.031**	**[0.11, 2.74]**
	Positive	Neutral	0.387	0.999	[−0.96, 1.06]
	Negative	Neutral	0.485	**0.030**	**[−2.65, −0.12]**
T5	Positive	Negative	0.416	0.149	[−0.23, 1.94]
	Positive	Neutral	0.292	0.984	[−0.86, 0.67]
	Negative	Neutral	0.434	0.115	[−2.08, 0.18]

Bolded data indicate *p* < 0.05.

### HRV

The SDNN and RMSSD descriptive statistics are displayed in Table 3.

**Table 3:** Descriptive statistics of SDNN and RMSSD (*M* ± *SD*).

HRV	T0	T1	T2	T3	T4	T5
SDNN_pos_	11.104 ± 4.135	4.684 ± 3.446	4.171 ± 6.012	4.079 ± 5.486	2.959 ± 1.020	4.974 ± 3.299
SDNN_neg_	16.164 ± 10.456	5.848 ± 6.609	3.561 ± 1.250	5.295 ± 10.436	6.938 ± 16.648	8.508 ± 9.858
SDNN_neu_	16.428 ± 11.683	5.864 ± 5.938	3.607 ± 1.066	3.508 ± 1.835	3.462 ± 1.838	5.514 ± 3.256
RMSSD_pos_	8.666 ± 4.023	4.183 ± 1.965	3.653 ± 1.786	3.343 ± 1.176	3.406 ± 1.385	4.017 ± 2.389
RMSSD_neg_	11.315 ± 4.905	4.320 ± 1.859	3.593 ± 0.961	3.963 ± 3.303	4.655 ± 6.163	6.005 ± 6.760
RMSSD_neu_	11.472 ± 5.622	4.270 ± 1.639	4.007 ± 1.515	4.183 ± 2.989	4.037 ± 2.648	4.354 ± 2.423

HRV, heart rate variability. SDNN = the standard deviation of normal-to-normal intervals. RMSSD = the root mean square of successive differences. SDNN_pos_, SDNN_neg_, and SDNN_neu_ indicate HRV measured when the question formulations were positive, negative, and neutral, respectively. RMSSD_pos_, RMSSD_neg_, and RMSSD_neu_ indicate HRV measured when the question formulations were positive, negative, and neutral, respectively.

**Figure 3:**
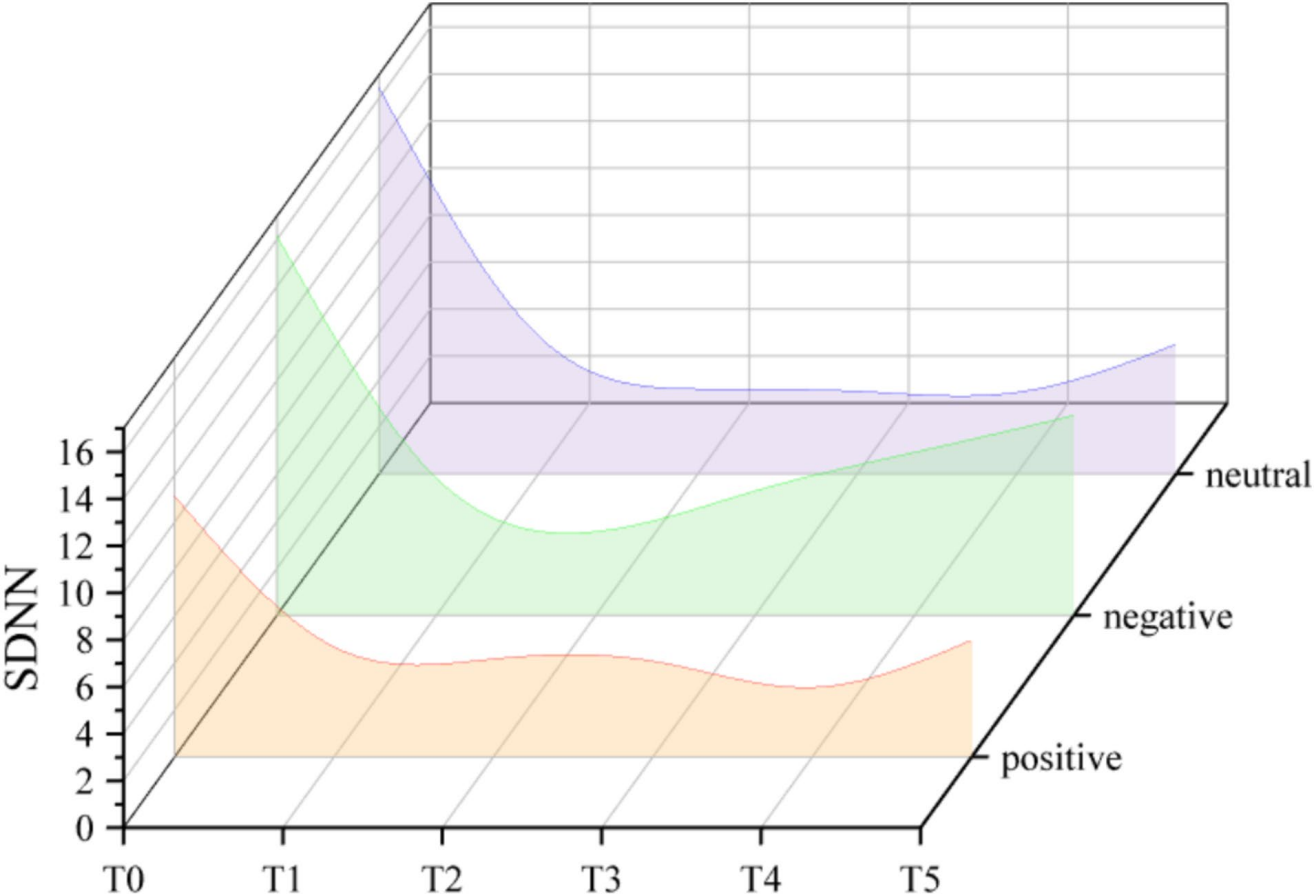
Trend of changes in SDNN under different question formulations.

When the dependent variable is SDNN (see Figure 3), the main effect of measurement time was significant (*F*_(2.514, 50.270)_ = 30.739, η*_p_*^2^ = 0.606, *p* < 0.001). When compared to 10 min of exercise, SDNN was much higher after 5 min (*p* < 0.05, 95% CI = [0.266, 3.105]). During the acute exercise, SDNN significantly decreased. In the meantime, SDNN was substantially lower following acute exercise than it was prior to exercise (T5 vs. T0: *p* < 0.001, 95% CI = [−10.861, −5.606]). However, the main effect of question formulation was not significant (*F*_(1.561, 31.223)_ = 2.381, η*_p_*^2^ = 0.106, *p* > 0.05), and the interaction was also not significant (*F*_(1.876, 37.523)_ = 1.060, η*_p_*^2^ = 0.050, *p* > 0.05).

**Figure 4:**
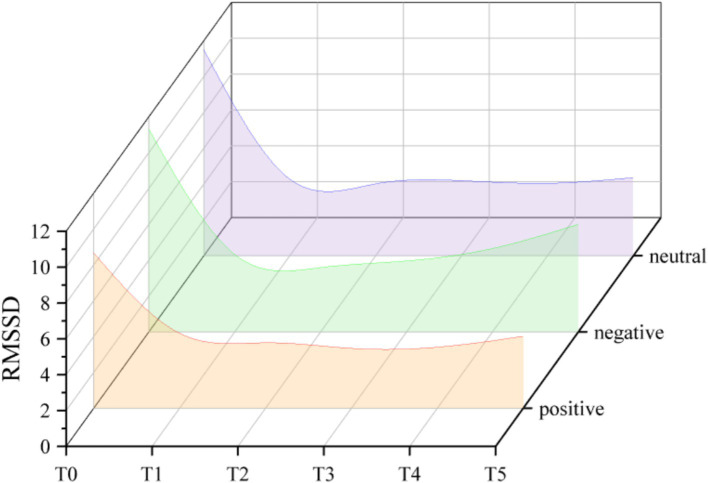
Trend of changes in RMSSD under different question formulations.

When the dependent variable is RMSSD (see Figure 4), the main effect of measurement time was significant (*F*_(2.115, 42.307)_ = 44.041, η*_p_*^2^ = 0.688, *p* < 0.001). When compared to 10 min of exercise, RMSSD was much higher after 5 min (*p* < 0.01, 95% CI = [0.139, 0.873]). During the acute exercise, RMSSD significantly decreased. Meanwhile, RMSSD was substantially lower following acute exercise than it was prior to exercise (T5 vs. T0: *p* < 0.001, 95% CI = [−7.334, −4.041]). However, the main effect of the question formulation was not significant (*F*_(1.567, 31.340)_ = 2.928, η*_p_*^2^ = 0.128, *p* > 0.05), and the interaction was also not significant (*F*_(2.019, 40.372)_ = 1.269, η*_p_*^2^ = 0.060, *p* > 0.05).

## Discussion

The current study developed positive and negative question formulations based on the neutral item of the Feeling Scale (FS). The objective was to examine potential variations in the impact of different question formulation measures on affective responses during acute exercise. In an effort to offer a psychophysiological explanation for affective responses under various question formulations, participants’ HRV was also measured. The results of the research indicate that varying question formulations exert an influence on affective reactions. The affective responses of individuals were found to be more positive when presented with a positive or neutral question formulation, as opposed to a negative question formulation. There was a rebound in the affective responses of individuals regardless of whether the question presented was neutral, positive, or negative. During acute exercise, participants’ HRV was indicated by a substantial decrease in both SDNN and RMSSD. The research hypothesis received partial support.

Participants will assess their current feelings while exercising in order to gather affective responses to various question formulations. Individuals reported greater favorable affective responses in our study when the question formulation was positive or neutral, as opposed to a negative question formulation. This supports the findings of Helfer et al. (2015), who found that more favorable affective responses result from positive induction. Over a prolonged period of time, the fatigue and muscular discomfort linked to exercise led to a shift in affective responses in a negative direction. As depicted in Figure 1, the mean values of affective responses exhibited a gradual decrease from T0 to T4 when negative and neutral question formulations were employed. However, when the question formulation was positive, a progressive rebound in affective responses occurred at T3. According to studies by Zenko et al. (2020), people who get prompts to maximize their enjoyment of exercise report feeling more joyous when exercising. The utilization of the term “pleasant” within the context of positive question formulation might serve as a favorable stimulus. Moreover, the implementation of a 5-min measure at consistent intervals contributes to a progressive enhancement of affective responses when employing the positive question formulation. Nevertheless, the impact of question formulations on affective reactions can only be observed during the duration of acute exercise. After exercising for 5 min, there was no apparent difference in the affective reactions to the various question formulations. The results of Cooper et al. (2022), who found that enjoyment increased individually between 5 and 20 min after exercise, are in line with this. This could be because energy was recovered and fatigue progressively subsided during the cool-down duration. Consequently, affective responses increased again.

Affective responses were less positive when the question formulation was negative compared to positive or neutral. It’s also consistent with earlier studies (Allen Catellier and Yang, 2013). When invited to watch either positive, negative, or neutral movie clips before exercising, Allen Catellier and Yang (2013) found that individuals with negative affect would be less likely to exercise than those with positive or neutral affect. This result can be explained by affective transfer (Isen, 2001), in which individuals frequently transmit their feelings from one object to another. It’s possible that the use of words like “bad” in the question formulation, which elicit negative affective responses, reduced participants’ enjoyment of the exercise in our study. This led to lower ratings for affective responses.

During acute exercise, there was a considerable decrease in HRV, which is in line with previous research (Rúa-Alonso et al., 2020). The outcomes also support the vagal tank theory (Laborde et al., 2018b). HRV increased throughout the warm-up, indicating that individuals’ behavioral and cognitive self-regulation have improved. As soon as the exercise began, HRV progressively dropped. This suggests that that individual experienced heightened sympathetic activation and diminished parasympathetic activity, which was further inhibited (Rajendra Acharya et al., 2006). However, the question formulation had no impact on the change in HRV. This phenomenon may be attributed to the inherent complexity of the systems and mechanisms underlying HRV, which arises from a combination of individual and environmental factors, along with their interactions (Laborde et al., 2018a). Alternatively, null findings in HRV studies may reflect the limited sensitivity of global metrics such as SDNN and RMSSD in capturing subtle cognitive-affective influences. The results further suggest that while question formulation does not affect the physiological measure of HRV, it does modulate an individual’s affective responses to psychological components.

This study has several limitations. Firstly, regular physical activity is beneficial to both physical and mental health (Lee et al., 2003; Warburton, 2006). However, our study did not explore exercise behavior directly. To ensure the rigor of the experimental data and control for extraneous variables, the current study was conducted within a laboratory setting. It is recommended that subsequent studies validate the stability of question formulations in naturalistic environments and track adherence to subsequent exercise. This approach would provide more practical intervention insights for promoting exercise behavior. Second, the gender gap and the fact that all participants were college students might have affected the findings’ external validity. Future research should aim to balance the gender ratio of the sample and collect relevant data such as participants’ physical activity levels. Analyzing the moderating effects of gender and physical activity level on the influence of question formulations on affective responses would enhance the ecological validity of the research. Thirdly, the intensity of exercise has the potential to impact affective responses (Ekkekakis et al., 2020). Future research could investigate the effect of question formulations on affective responses under varying exercise intensities (e.g., high-intensity exercise). Fourth, this study lacked manipulation checks for the question formulations. Subsequent research should incorporate manipulation checks for the question formulations, potentially using Likert scales or lexical emotional intensity analysis.

## Conclusion

Affective response is one of the most important predictors of future physical activity (Williams et al., 2012). Improving a person’s affective responses to exercise is essential. When measured using positive or neutral question formulations, individuals’ affective responses were more positive. Specifically, positive or neutral question formulations may generate favorable reactions, leading to higher positive affective responses in exercise. This approach offers an easy way to enhance affective responses. To improve people’s affective experiences and encourage healthier behavior, additional research into the underlying mechanistic difficulties is still required.

## Data Availability

The raw data supporting the conclusions of this article will be made available by the authors, without undue reservation.
